# Fluctuation and extinction of laminar diffusion flame induced by external acoustic wave and source

**DOI:** 10.1038/s41598-021-93648-0

**Published:** 2021-07-13

**Authors:** Caiyi Xiong, Yanhui Liu, Haoran Fan, Xinyan Huang, Yuji Nakamura

**Affiliations:** 1 Research Centre for Fire Safety Engineering, Department of Building Services Engineering, The Hong Kong Polytechnic University, Kowloon, Hong Kong China; 2 The Hong Kong Polytechnic University Shenzhen Research Institute, Shenzhen, China; 3 Department of Mechanical Engineering, Toyohashi University of Technology, Toyohashi, Japan

**Keywords:** Mechanical engineering, Fluid dynamics

## Abstract

Acoustic wave can destabilize the flame and has a potential in firefighting, but the influences of the sound source and its frequency are still poorly understood. This work applies a loudspeaker to extinguish a laminar diffusion propane flame of 5–25 mm high, where the local sound frequency is 50–70 Hz and sound pressure is 0.8–3.2 Pa (92.0–104.1 dB). Results reveal a constant flame pulsating displacement at the extinction limit, independent of the sound environment used. Such a flame pulsating displacement is found to be caused by the motion of the speaker membrane (or diaphragm) and its induced wind, which could be two orders of magnitude larger than the displacement of the air that transmits acoustic wave. Thus, under the influence of sound source, a critical flame strain rate, stretched by the pulsating airflow, can be formulated to characterize the blow-off limit better than the local sound pressure. The sound source with a lower frequency can produce larger pulsating displacements of both membrane and flame, and thus promoting extinction. This work improves the understanding of flame dynamics under the external sound field and source, and it helps establish a scientific framework for acoustic-based fire suppression technologies.

## Introduction

Fire suppression plays a central role in fire protection and active firefighting^1–3^. Today, the combustion and fire science community is continually searching for more effective and flexible fire suppression technologies^3–5^. One proposing approach is the use of acoustic waves to destabilize and extinguish the diffusion flame^6–13^, because the flame is sensitive to the airflow field. Nevertheless, different from the external airflow (or wind), the acoustic-induced flow can create a quick fluctuation in the flame sheet, because the nature of sound is a longitudinal wave.

The response of flame to the external sound field is affected by the sound frequency^6–15^. When using a high sound frequency (> 200 Hz), it is difficult to extinguish a flame^16–18^ but can only cause flame tilting or fluctuation^13^. Several theoretical and experimental studies investigated the flame stabilization in a sound filed, particularly for the coupling of acoustic and droplet combustion and for different engine applications^19–23^. In contrast, the low-frequency sound, which shows excellence in destabilizing the flame, was rarely investigated for its potential application in fire suppression.

McKinney and Dunn-Rankin^9^ first used the low-frequency acoustic waves (75–135 Hz) to extinguish a methanol droplet flame. They found that a higher sound pressure was required to extinguish the flame at a higher frequency and concluded that the extinction was caused by the acoustic-induced flame displacement from the fuel droplet. The US Defense Advanced Research Projects Agency (DARPA)^6^ then systematically investigated the acoustic extinction of a 15-cm non-premixed methane flame, where the sound used had a frequency ranging from 35 to 150 Hz. The positive correlation between the extinction sound pressure and frequency was again verified. More importantly, the extinction condition was found to be independent of the burner size, implying that the sound-based flame suppression technology has the potential to work for larger flames. Fredman and Stoliarov^7^ examined the acoustic extinction of liquid-fuel flames using the sound with a frequency of 30–50 Hz and pressure of 5–50 Pa and compared with the flame extinction by a fan-driven flow. Niegodajew et al*.*^8^ further used a schlieren apparatus to observe the acoustic-induced extinction process. It is worth noting that all the above studies used cylindrical tubes or collimators to converge and intensify acoustic waves, and the flames tested were all very close to the free end of the tubes used (≤ 60 mm). However, the influence of such an intensified sound source is not well understood.

Our recent works used a loudspeaker and low-frequency acoustic waves (without tube between speaker and flame) to extinguish the stationary candle flame^14^, the moving flames in plastic drips^14,24^, and the flaming firebrand^15^. By converting the sound pressure into a velocity component, a characteristic Damköhler (Da) number was introduced as an indicator for the acoustic extinction limit. More recently, Yamazaki et al*.*^13,25^ found that the flame motion in front of the speaker was likely caused by the periodic motion of the speaker membrane (cone or diaphragm) rather than the acoustic pressure. Thus, two questions remain, (1) *why a lower-frequency sound shows a better flame-extinction performance?* and (2) *what is the influence of sound source on acoustic-driven flame extinction?*

In this work, the target flame chose a buoyancy-driven laminar diffusion flame with heights of 5–25 mm, produced by a propane gas burner. The sound fields with a frequency of 50–70 Hz and pressure of 0.8–3.2 Pa were produced by a large speaker with a membrane diameter of 460 mm but without the tubular sound amplifier. The displacements of the flame, speaker membrane, and gas particles were quantified, and a comprehensive analysis based on both flame dynamics and acoustic theory was performed to answer these two questions.

## Experimental methods

### Target flame

**Figure 1:**
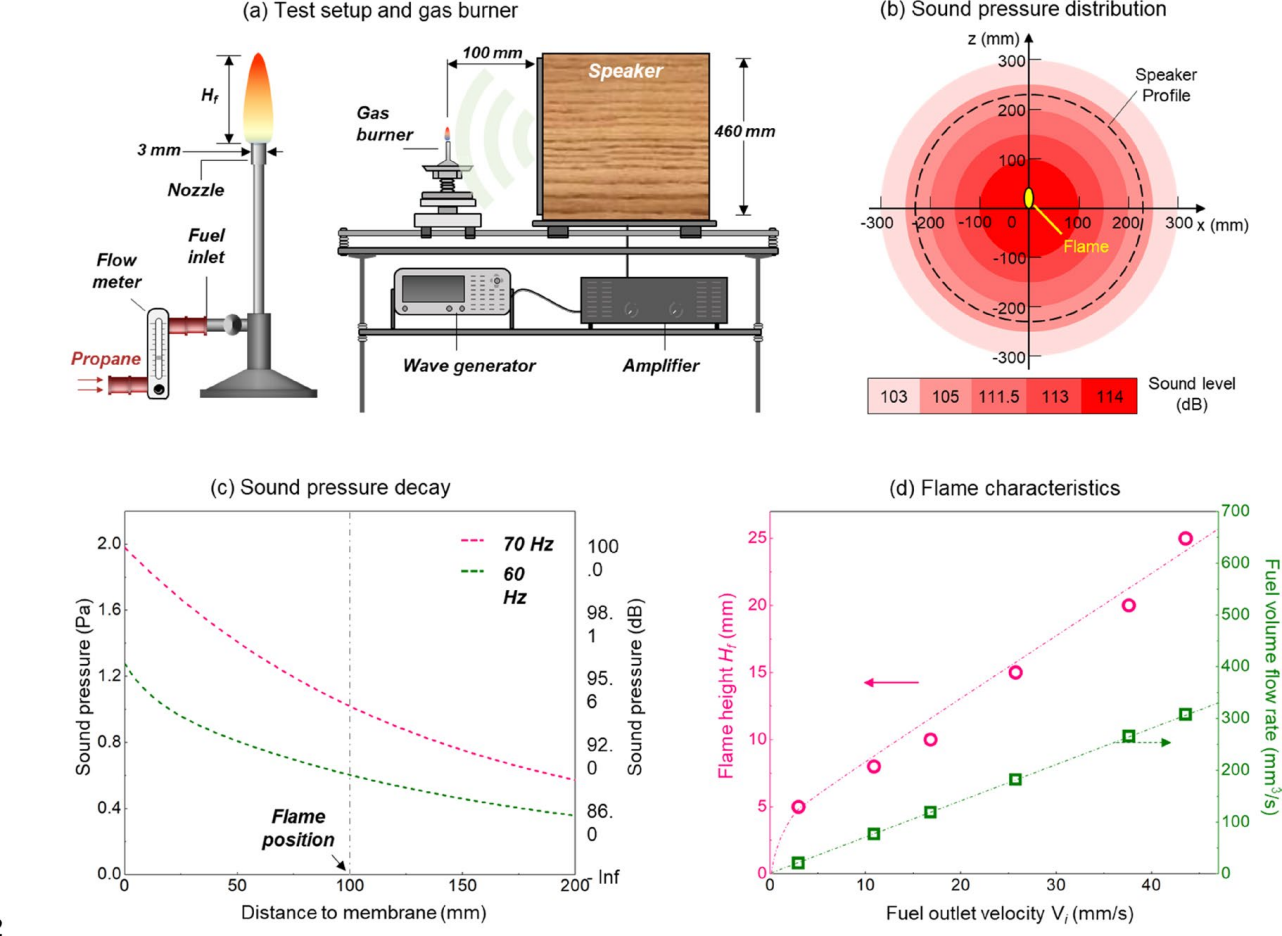
(**a**) The schematic of gas burner and sound source, (**b**) sound pressure distribution on the plane 100-mm away from the speaker^15^, (**c**) the decay of sound pressure with the distance to the speaker membrane, and (**d**) the corresponding flame characteristics.

A gas burner shown in Fig. 1 was employed in front of a loud speaker and sound field to produce diffusion flames, where the nozzle had a diameter of 3 mm. The fuel inlet on the burner was connected to a gas tank filled with pure propane. A rotameter (LZB-3WB) with an accuracy of 1.6% F.S. (Full Scale: 0.06 LPM) was used to control the fuel flow rate. The target flame was studied at different heights from 5 to 25 mm, which helps explore the interplay between acoustic and flame and reveals the mechanism behind acoustic extinction. The dependences of flame height (${H}_{f}$) and fuel volume flow rate on the fuel injection velocity (${V}_{i}$) were shown in Fig. 1d. The key parameters for each flame were listed in Table 1. In general, each flame has a Froude number (${F}_{r}={V}_{i}^{2}/g{H}_{f}$) less than 0.01, and their Grashof numbers are less than 10^5^. Thus, all tested diffusion flames are unlifted and laminar, driven by the buoyancy.

**Table 1:** Properties and thermophysical parameters for all target flames.

${H}_{f}$[mm]	${V}_{F}$[mm/s]	${\dot{Q}}_{F}$[mm^3^/s]	HRR (W)	$Re$	$Fr$	$Gr$
5	3.0	21	2.1	0.3	0.002	3.8 × 10^2^
10	16.8	119	11.7	3.9	0.003	3.0 × 10^3^
15	25.7	182	17.9	8.9	0.005	1.0 × 10^4^
20	37.6	266	26.1	17.4	0.007	2.4 × 10^4^
25	43.6	308	30.3	25.2	0.008	4.7 × 10^4^

### External sound source and field

Figure 1a also shows the experimental set-up for producing a uniform external sound field. A wave generator was used to produce the initial signal, which mainly controlled the sound frequency. An amplifier was then employed for signal enhancement, which primarily controlled the sound pressure. Finally, a speaker was responsible for transferring the signal to a uniform sound field. The burner was fixed in the near field from the speaker and is 100-mm in front of the speaker with the nozzle leveling with the speaker center. The diameter of the speaker membrane (cone or diaphragm) was 460 mm and was much larger than the size of the flame (≤ 25 mm). This configuration can help simplify the spherical waves from the speaker as a semi-1D wave at the flame, given the degree of asymmetry of the source and the proximity of the flame to the speaker.

More importantly, there was no tubular sound amplifier or sidewall nearby the speaker to concentrate the sound wave, different from past studies^6–9^. Thus, the sound field around flame was almost homogeneous, as quantified previously^14,15^ and seen in Figs. 1b–c.

Based on the extinguishing excellence of the low-frequency sound^14^, a frequency band varying from 50 to 70 Hz was selected. This frequency was slightly higher than the band from 30 to 50 Hz used in other experiments^7,8^, where their sound fields were all amplified by a cylindrical tube. As such, the sound field used in this work was free to develop rather than being intensified as before, and thus a much higher speaker power was required for extinction. More importantly, the resistance of the speaker decreases with the decrease of its working frequency, so further decreasing the sound frequency to lower than 50 Hz may damage the speaker via a short circuit. A free-field decibel meter TES-1352S was employed to measure sound pressure, which works from 30 to 130 dB with an accuracy of 0.1 dB ($\text{dB}=20\mathrm{l}\mathrm{g}[Pa/(2\times {10}^{-5})]$ and see Fig. 1b). During the experiment, the decibel meter was placed at the flame position with its transducer facing the speaker diaphragm.

### Image processing for displacement

**Figure 2:**
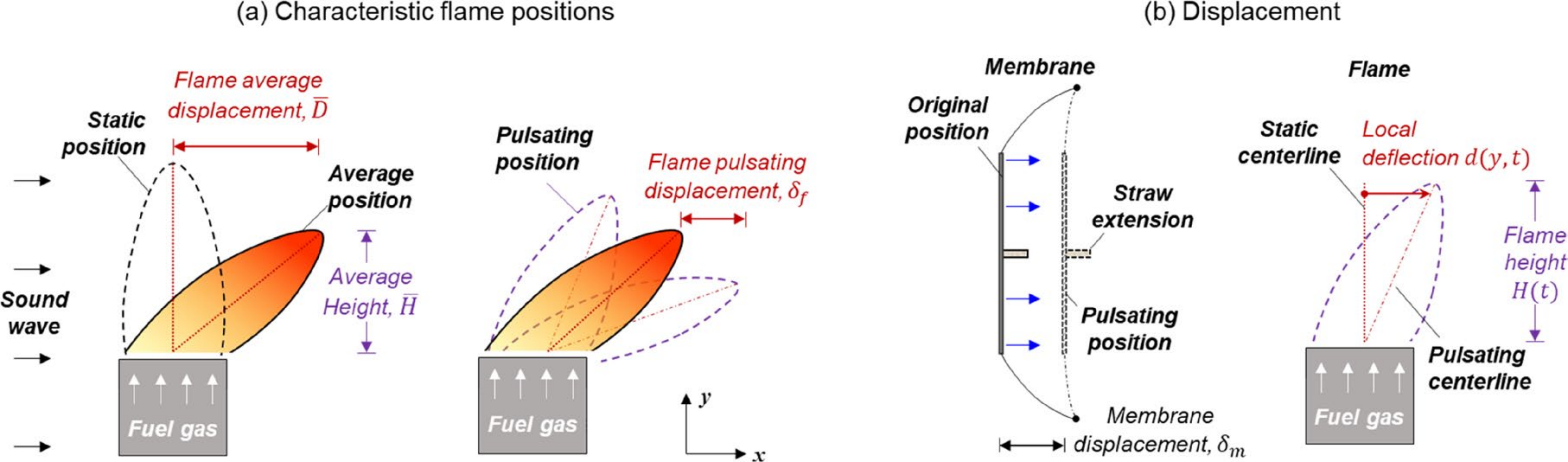
(**a**) The characteristic flame positions during fluctuation, and (**b**) displacements of speaker membrane (straw) and flame.

A high-speed camera with a shutter speed of 1,000 fps was used to monitor the motions of flame (Videos S1-2) and speaker membrane (Videos S3), where the resolution per pixel is 0.03 mm/pixel. Several flame positions are defined for image processing, seen in Fig. 2a. Initially, without the activation of the speaker, the flame is purely buoyancy-driven and laminar at the ‘static position.’ When the speaker is activated, the flame will be first deflected to an ‘average position,’ depending on the initial condition in contact with the sound field. The further fluctuation of flame around its ‘average position’ is caused by the external sound source, and the instantaneous flame position during the fluctuation is termed as ‘pulsating position.’

To facilitate the image processing, a Cartesian x–y coordinate was utilized, which helps describe the flame displacement. The flame inclination and displacement change with its height ($\overrightarrow{y}$). Therefore, two spatial and temporal average values are used to characterize the flame displacements at the wave propagation direction ($\overrightarrow{x}$), i.e., (1) the average static horizontal displacement from the static centerline, $\bar{D}$ (see Fig. 2a), and (2) the average pulsating displacement around its average position, ${\delta }_{f}$ (see Fig. 2a).

Calculations of both flame displacements were conducted based on Fig. 2b, where the average flame displacement ($\bar{D}$) can be given by the spatial and temporal average of the local flame deflection ($d\left[y\left(i\right),t\right]$ in Fig. 2b) as

1a $$\bar{D} = {\left. {\mathop \sum \limits_{j = 1}^M \frac{{D\left[ {t\left( j \right)} \right]}}{M}} \right|_{t\left( 1 \right) = 0,\;t\left( M \right) = {t_o}}}$$

1b $$D\left( t \right) = {\left. {\mathop \sum \limits_{i = 1}^N \frac{{d\left[ {y\left( i \right),t} \right]}}{N}} \right|_{y\left( 1 \right) = 0,\;\;y\left( N \right) = H}}$$

Here, $N$ is the number of vertical pixels of the flame image, $H$ is the instantaneous flame height, and ${t}_{0}$ = 2 s is the duration of high-speed video. Also, the flame pulsating displacement (${\delta }_{f}$) can be given by the standard deviation of $D\left(t\right)$:

2 $${\delta_f} = {\left. {\sqrt {\frac{{\mathop \sum \nolimits_{j = 1}^M {{\left( {D\left[ {t\left( j \right)} \right] - \bar D} \right)}^2}}}{M}} } \right|_{t\left( 1 \right) = 0,\;t\left( M \right) = {t_o}}}$$

where $M$ is the total frame number of the high-speed video. All equations were solved via an in-house MATLAB code during video processing.

A similar approach was used to quantify the displacement of the speaker membrane (Fig. 2b). Considering that the membrane was regressed into the enclosure and it was difficult to measure its displacement directly, a light plastic straw of 100 mm in length was horizontally glued to the membrane as an indicator, since it could fluctuate in a good rhythm and amplitude with the speaker membrane. Video S3 in the supplemental material shows the motion of straw (membrane). In this way, the motion of the straw’s free end can be monitored by the high-speed camera and measured by the same code.

### Experimental procedure

Since the stability of the laminar flame is susceptible to the temperature of the burner^26^, the flame would first burn for 5 min before each experiment to ensure a stable temperature on the burner nozzle, monitored by a thermocouple. For each test, a sound field with specific frequency and pressure was first generated, and there was a soundproofing panel to separate the flame from the sound field. Then, the gas burner was moved to its designed position. After removing the panel slowly, a successful extinction was defined as the one occurred immediately. By a stepwise increase or decrease of the sound pressure, a critical extinction limit could be determined. After extinction, the measurement on the displacement of speaker membrane was conducted at the same sound environment, with the use of the straw extension. All experiments were repeated three times to reduce the random error and calculate the uncertainty.

## Results and discussion

### Near-limit flame behaviors

**Figure 3:**
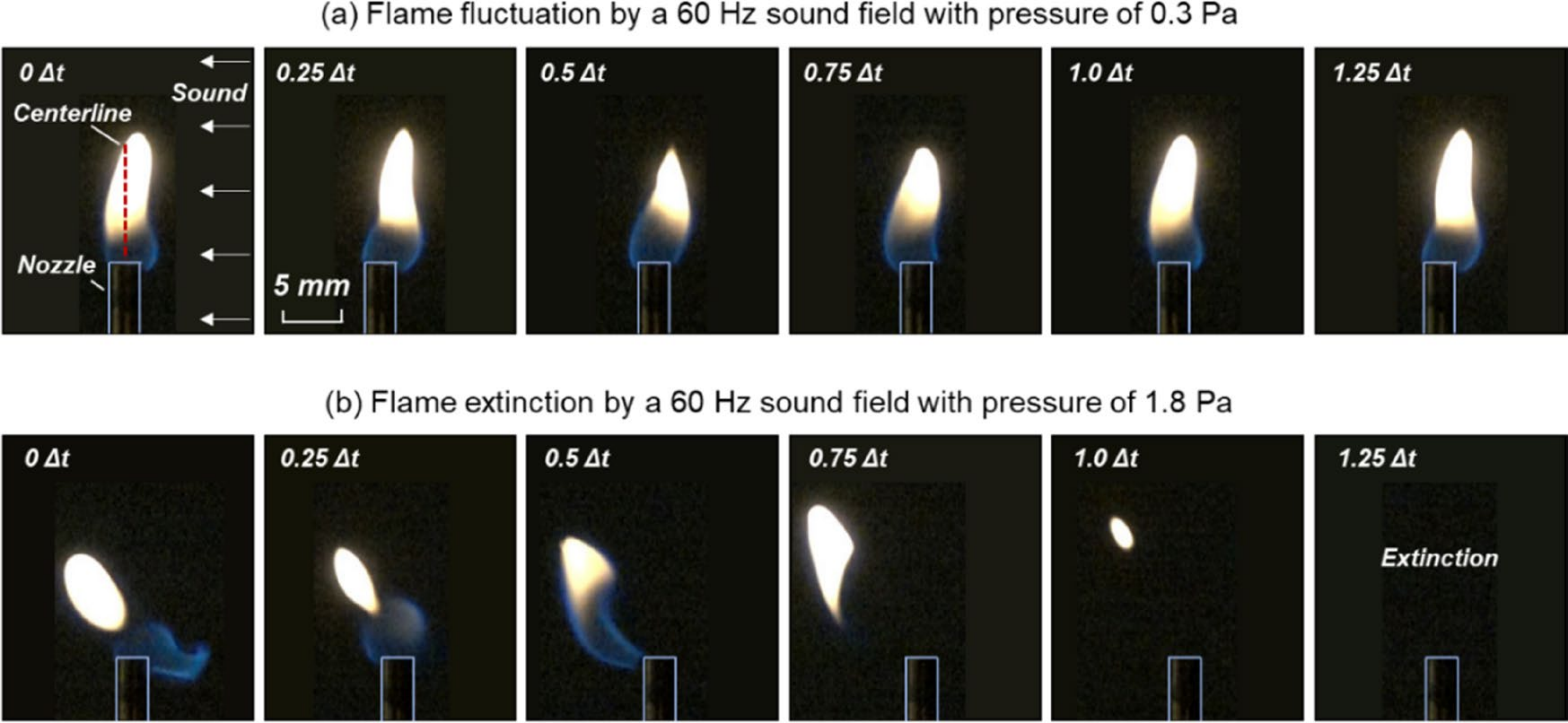
Behaviors of a 15-mm flame inside a 60-Hz sound field with a pressure of (**a**) 0.3 Pa (Video S1) and (**b**) 1.8 Pa (Video S2), where Δt = 16.7 ms is the acoustical cycle.

Figure 3a shows a typical fluctuation process of a 15-mm flame caused by sound, where the speaker emitted 60-Hz acoustic waves from the right-hand side (RHS), and the local sound pressure was 0.3 Pa at the flame position. Referring to the fixed position of the burner nozzle, the flame kept fluctuating between right and left, parallel to the direction of wave propagation. Image analysis confirmed that the flame fluctuation was strictly synchronized with the acoustic cycle (${f}_{f}={f}_{s}$). When increasing the sound pressure to 1.8 Pa (or 99 dB), a successful extinction of a 15-mm flame can be seen in Fig. 3b, where the flame fluctuation still existed, but the flame was deflected far away from the nozzle and could no longer be anchored, and eventually, acoustic extinction occurred.

**Figure 4:**
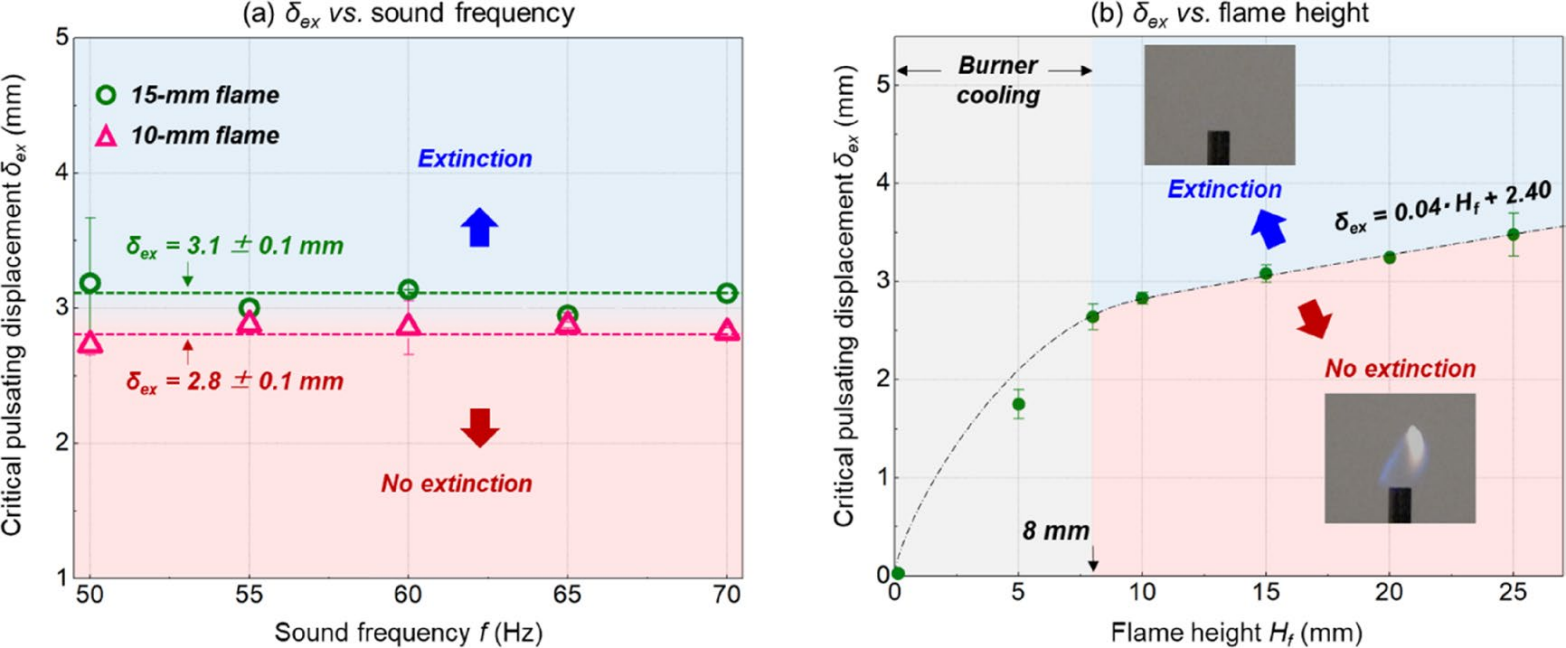
Dependence of critical pulsating displacement on (**a**) sound frequency and (**b**) flame height.

Imaging analysis also showed that the average flame displacement ($\bar{D}$) in Eq. (1a) was random in repeating tests, caused by a random initial phase when the flame was in contact with the sound. By contrast, the flame pulsating displacement at extinction limit, namely ${\delta }_{ex}$, could be a stable extinction indicator. Figure 4a shows that extinction must occur once ${\delta }_{f}={\delta }_{ex}$, regardless of the sound frequency and pressure.

Figure 4b further shows that ${\delta }_{ex}$ increases linearly with the flame height (${H}_{f}$) as

3 $${\delta_{ex}} = 0.04{H_f} + 2.4$$

where the units of ${\delta }_{ex}$ and ${H}_{f}$ are mm, and the flame height should be larger than 8 mm. That is, if the flame height increases 1 mm (or HRR increases 1.3 W), the critical pulsating displacement ${\delta }_{ex}$ should increase at least 0.04 mm to cause extinction. Such a correlation may help explain the mechanism of extinction under the external sound field. Note that for the flame smaller than 8 mm, the cooling effect by the burner itself may dominate flame extinction.

### Extinction mechanism

**Figure 5:**
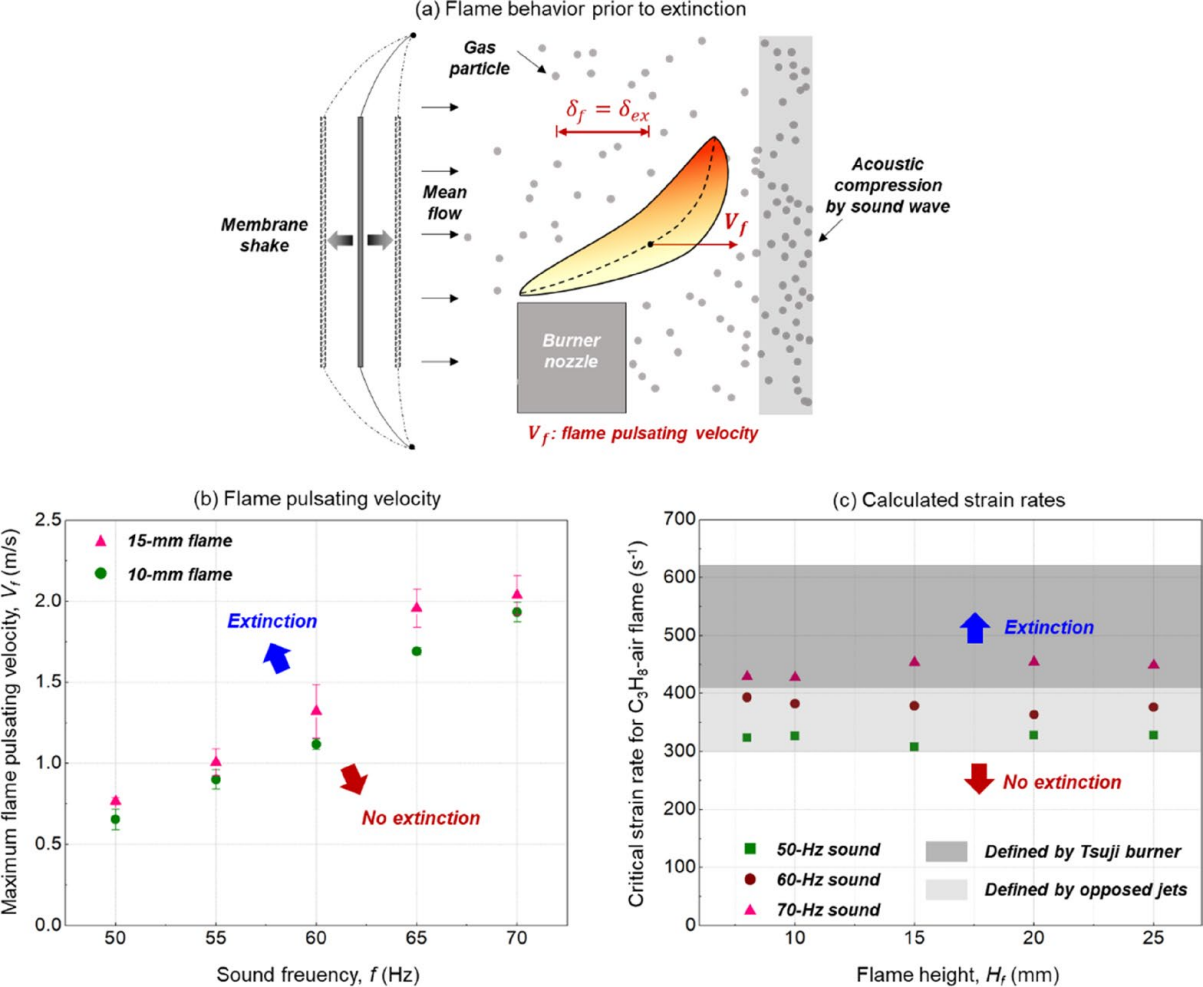
(**a**) Illustration of the near-limit flame shape, (**b**) the maximum flame pulsating velocity, and (**c**) critical strain rates at the extinction limits.

Experiments suggested that a necessary condition for acoustic extinction is that the flame should be deflected to a distance far from the nozzle. This could be explained by a critical Damköhler (*Da*) number, when the flow time scale of flame in the reaction zone becomes short, compared to the chemical time scale. In this way, a critical strain rate ($\alpha$) can be defined for the extinction limit,

4 $$\alpha = \frac{V_f}{\delta _{ex}}$$

where the maximum flame pulsating displacement (${\delta }_{ex}$) was selected as the characteristic radius. Note that the fuel injection velocity (${V}_{i}$) is not used in Eq. (4) because the flame is driven by buoyancy instead of fuel jet. As a result, the maximum flame pulsating velocity (${V}_{f}$) in the wave direction is considered the only factor for stretching flame, obtained from the motion of flame centerline via image analysis. Figure 5a illustrates the flame stretch near extinction, which is like a classical counterflow flame. Figure 5b shows the variation of ${V}_{f}$ at the extinction limit versus the sound frequency.

Figure 5c further shows that at a fixed sound frequency, the critical strain rate remains constant for flames with different heights. Despite varying the sound frequency, all calculated strain rates were close to those found in classical counterflow propane-air flames, such as the Tsuji burner^27^ and the opposed jets^28–30^. Therefore, the critical strain rate, as well as the linear correlation between critical flame displacement and height (${\delta }_{ex}$ & ${H}_{f}$) in Fig. 4b, can indicate the flame extinction limit. On the other hand, the positive correlation between the critical strain rate and sound frequency was also reported previously^9^, where the acoustic extinction of tiny droplet flame was studied. It is possible that the diffusion velocity within the flame sheet should also be considered, which requires a more detailed analysis of the coupled flame-acoustic field in future study.

### Flame motion vs. sound field

**Figure 6:**
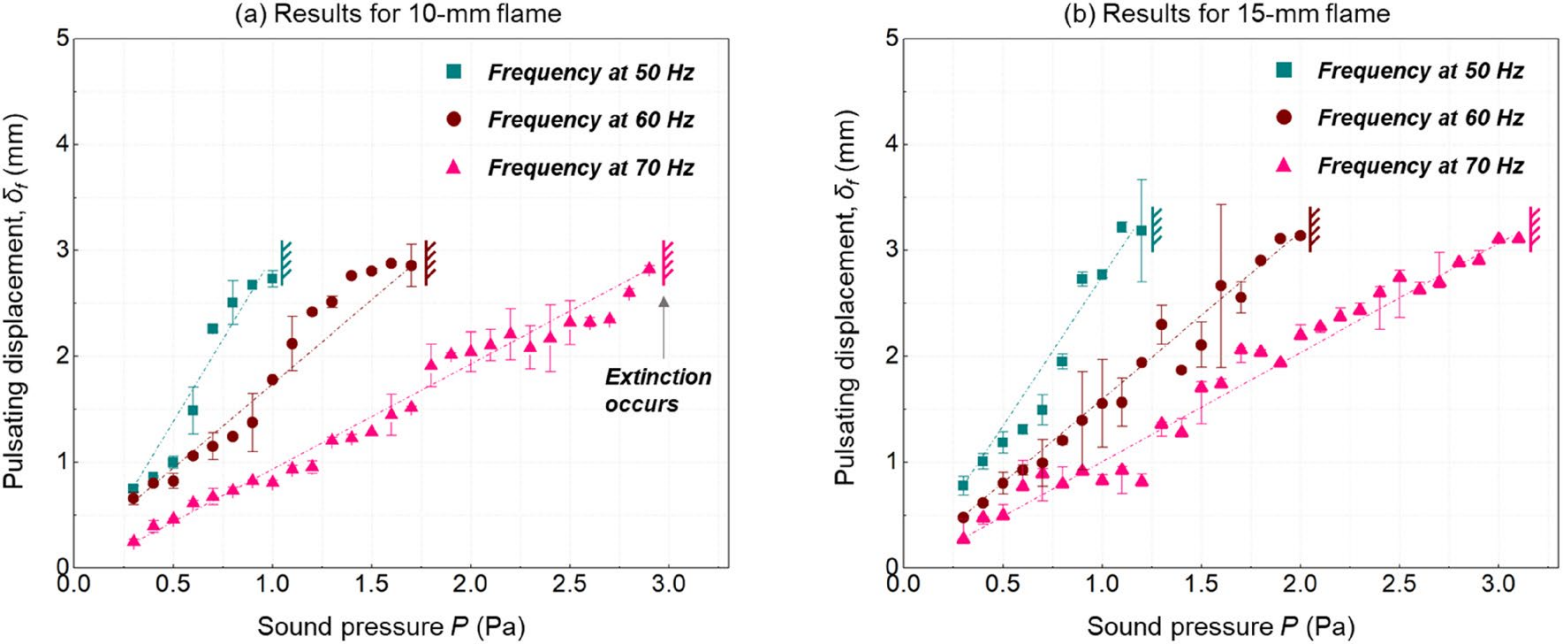
Dependence of flame pulsating displacement on sound pressure for (**a**) 10-mm and (**b**) 15-mm flames under different sound frequencies, where the error bar shows the uncertainty of repeating tests.

The flame pulsating displacement (${\delta }_{f}$) can also connect the near-limit flame dynamics with the sound field. Before extinction, the flame will only fluctuate at the frequency same as the sound source (Video S2). Figure 6 shows the dependence of ${\delta }_{f}$ on sound pressure $P$ at varying frequencies. Although the critical value (${\delta }_{ex}$) remains constant at extinction, there is a linear correlation between ${\delta }_{f}$ and $P$ as

5 $${\delta}_{f}\propto P$$

Moreover, ${\delta }_{f}$ increases faster under a lower sound frequency. In other words, with the use of a lower sound frequency, it is easier for ${\delta }_{f}$ to reach the extinction limit ${\delta }_{ex}$ by imposing a smaller pressure increment. Consequently, a lower-frequency sound can show a better flame-extinction performance. Note that the pressure in Pa here was converted directly from the SPL in dB.

**Figure 7:**
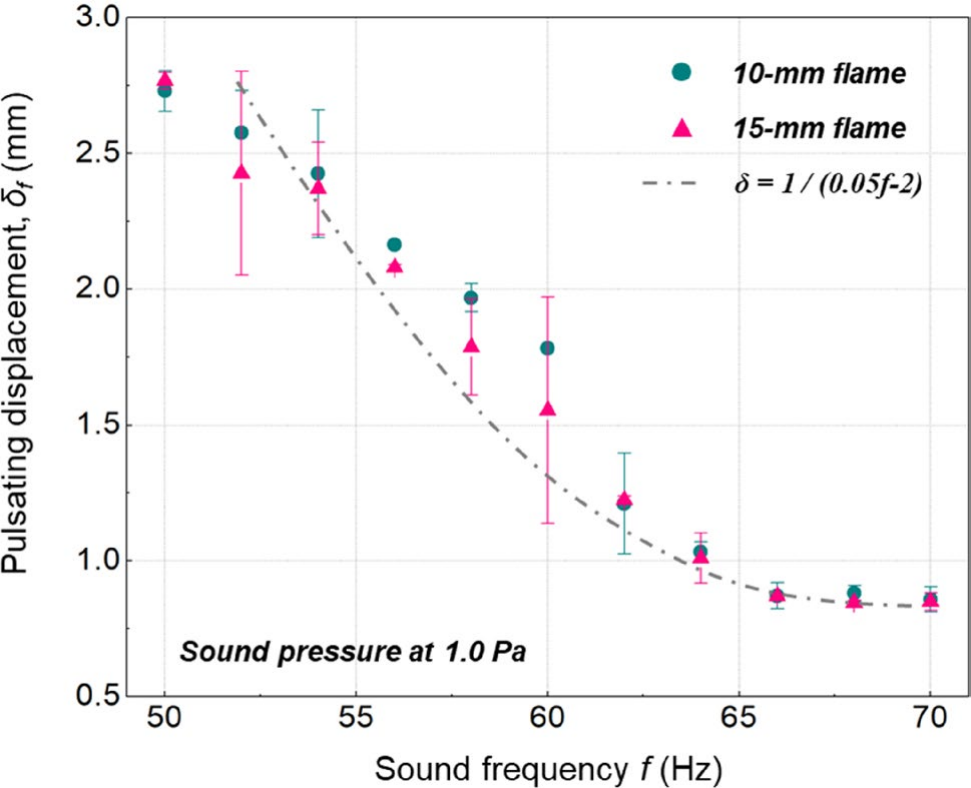
Dependence of flame pulsating displacement on sound frequency.

The dependence of flame pulsating displacement ${\delta }_{f}$ on the sound frequency $f$ was also explored. Here, the target flames were placed in the sound fields with frequency increasing from 50–70 Hz with a step of 2 Hz, while the sound pressure was kept constant at 1.0 Pa. Figure 7 indicates that as the $f$ increases, ${\delta }_{f}$ will decrease in a hyperbolic manner. Together with Eq. (5), we can get

6 $${\delta _f} \propto \frac{P}{f}$$

where a conventional hyperbolic fitting gives an excellent agreement, as shown in Fig. 7.

On the other hand, it is the fluctuation of air molecule to transmit the acoustic wave, and the transient air molecule displacement ($\sigma$)^31,32^ and amplitude ($\bar{\sigma}$) can be calculated based on the near-field plane wave assumption:

7a $$\sigma = \frac{P}{{2\pi fZ}}\cos \left( {\vec k \cdot \vec r - \omega t + {\varphi _0}} \right) \propto \frac{P}{f}$$

7b $$\bar{\sigma}=\frac{P}{2{\pi}fZ}$$

where $Z$ denotes the specific acoustic impedance of air, $\overrightarrow{k}$ denotes the angular wavevector, $\overrightarrow{r}$ is the position vector, $\omega$ is the angular frequency, and ${\varphi }_{0}$ is the initial phase of the sound wave. Note that $Z=$ 420 Pa∙s/m at room temperature and 223 Pa s/m at 1000 K^31,32^ (the average of room temperature and propane-air flame temperature).

**Figure 8:**
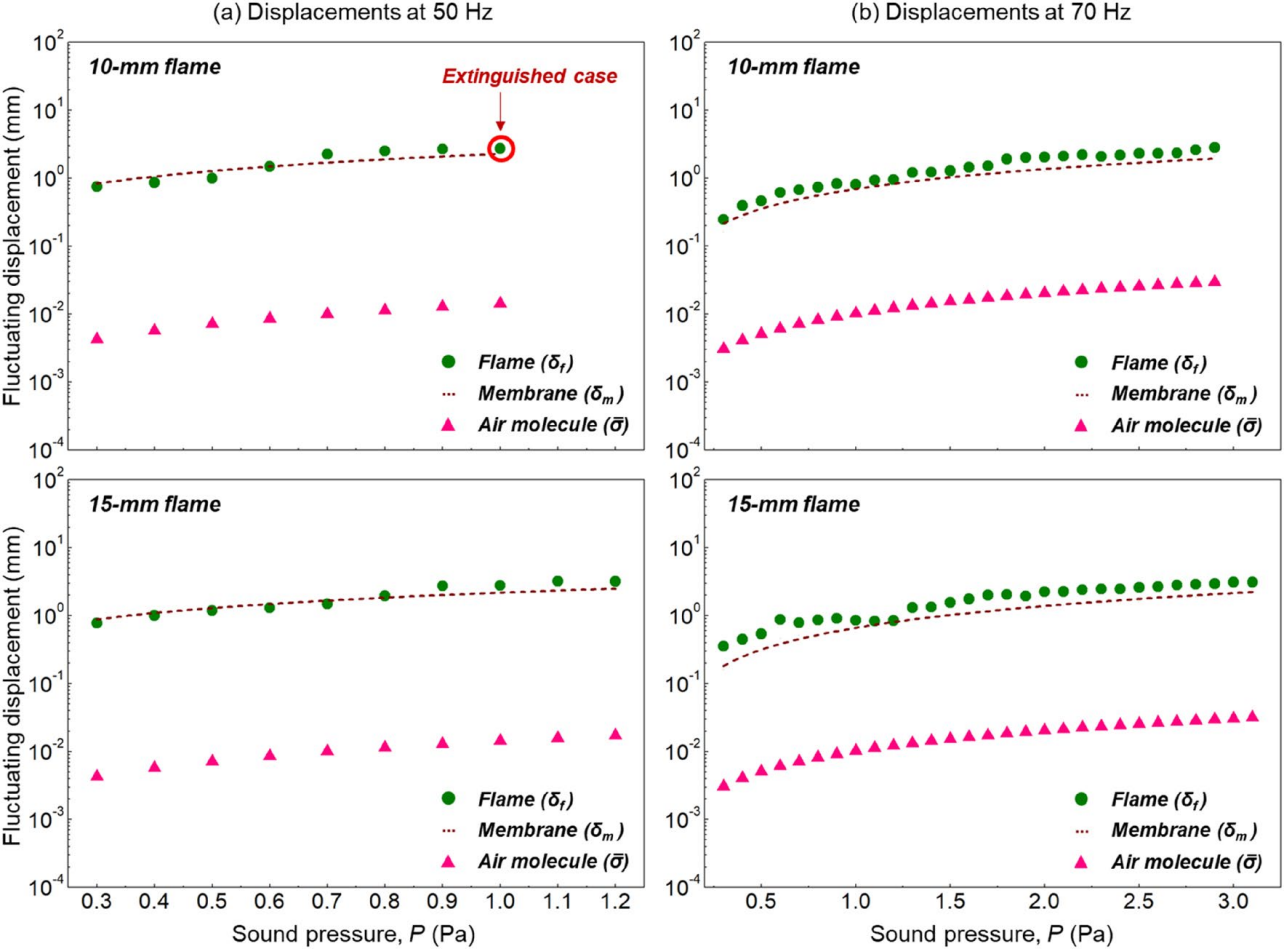
Comparison of the displacements of flame, speaker membrane, and air molecule at the sound frequency of (**a**) 50 Hz and (**b**) 70 Hz.

Comparison between Eqs. (6) and (7a) showed that the displacements of flame (${\delta }_{f}$) and air molecule ($\bar{\sigma }$) have a similar mathematical expression, i.e., ${\delta }_{f} \sim \bar{\sigma }\propto \frac{P}{f}$. Then, it is necessary to compare the magnitudes of these two displacements. Figure 8 shows a comparison between ${\delta }_{f}$ and $\bar{\sigma }$ under different flame heights and sound fields, where $\bar{\sigma }$ is given by Eq. (7b) with $Z=$ 223 Pa∙s/m. Clearly, the displacement of the air molecule is much smaller than the observed flame displacement.

**Figure 9:**
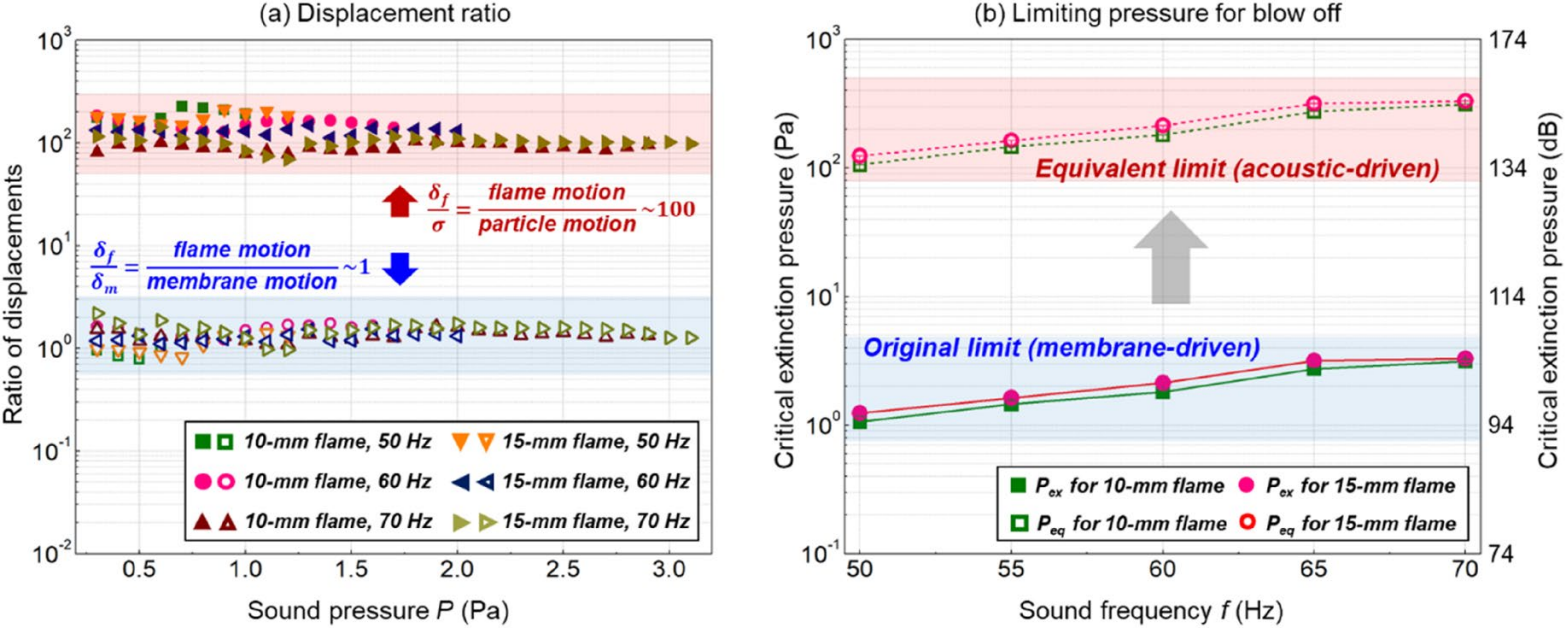
(**a**) Ratio of displacements, and (**b**) the original and equivalent extinction limits, where the conversion of sound pressure unit is $\text{dB}=20\bullet \mathrm{l}\mathrm{g}[Pa/(2\times {10}^{-5})]$.

Figure 9a further confirms that the flame pulsating displacement (${\delta }_{f}$) is about two orders of magnitude larger than the acoustic-driven air molecule displacement $\bar{\sigma }$

8 $$\frac{{\delta }_{f}}{\bar{\sigma }}=\frac{{P}_{d}}{{P}_{s}}\sim {10}^{2}$$

where the local dynamic pressure (${P}_{d}$) and sound pressure (${P}_{s}$) should be proportional to their displacements under the same sound frequency. Thus, it can be concluded that the motion of air molecule induced purely by the acoustic wave is not the main contributor to the fluctuation and extinction of the flame, although they have the same dependence on the sound field. There must be other factors that dominate the flame pulsating motion and extinction.

### Flame motion vs. membrane motion

Reexamining the sound source in Fig. 1a, a speaker is responsible for transferring the electrical signal to sound, where its membrane fluctuates at the same frequency as the signal. The membrane compresses the air and guides the sound wave to a specific direction, as seen in Fig. 5a. Since the speaker membrane motion could also generate an air motion, it is necessary to check whether the membrane contributes to flame motion.

Figure 8 also shows the displacement of the speaker membrane (${\delta }_{m}$ measured by straw extension) at different sound fields. There is a good agreement between the displacements of flame and membrane

9 $${\delta }_{f}\approx {\delta }_{m}$$

which is verified by their ratio (${\delta }_{f}/{\delta }_{m}\approx 1$) in Fig. 9a. Because the pulsating frequency of the membrane and the flame are the same, their maximum pulsating velocities are the same as well. Results evidence that the membrane of the speaker should be the main contributor to the fluctuation and extinction of flame. This is also the reason why the observed “acoustic-induced” flame inclination and extinction in some past studies is similar to the blow-off by external wind (or fan)^7,25^.

As such, in the near field of a membrane-based speaker, or in the sound field converged by a cylindrical tube or collimator as^7–9^, the net airflow can be a combination of two flows:

(1) an incompressible mean flow (like an external wind), produced by speaker membrane and indicated by the dynamic pressure ${P}_{d}$, and

(2) a compressible fluctuating flow, produced by sound waves propagation and indicted by the sound pressure ${P}_{s}$.

Both flows decrease when moving away from the speaker^25^, indicated by the decrease in both dynamic pressure and sound pressure (Fig. 1c). In general, the membrane-induced mean flow decays due to spatial dissipation, so it dominates the local flow mainly in the near field. In the current work, the target flame was close to the membrane (100 mm), where the decay of mean flow was small. As expected, increasing the distance between flame and speaker, flame extinction becomes more difficult. It is therefore many past studies used a cylindrical tube^6–9^ to facilitate acoustic extinction by reducing and guiding the spatial dissipation of the membrane-induced flow.

As discussed above, the membrane-induced mean flow is responsible for flame fluctuation and extinction. Hence, the local sound pressure (${P}_{ex}$) may not be an ideal extinction indicator. Assuming an idealized sound field without the influence of the membrane-induced mean flow, what is the equivalent sound pressure that enables the displacement of air molecule to exceed the critical flame pulsating displacement and cause blow-off? Based on Eq. (8), this equivalent sound pressure (${P}_{eq}$) is

10 $$\frac{{P}_{eq}}{{P}_{ex}}=\frac{{\delta }_{ex}}{\bar{\sigma }}\approx \frac{{\delta }_{f}}{\bar{\sigma }}\sim {10}^{2}$$

which could be at least two orders of magnitude greater than the local sound pressure.

Based on this, both the original and equivalent extinction limits for the 10-mm and 15-mm flames were presented in Fig. 9b. Specifically, an equivalent sound pressure (${P}_{eq}$) of 100–300 Pa (134–144 dB) is required to acoustically extinguish the target flames. For reference, a sound pressure of 140 dB is equivalent to the sound of firecrackers, airplane takeoff heard from about 50 m, or a gunshot from 30 m, which will cause unbearable pain to human beings^33^. However, the use of a cylindrical tube can effectively avoid such a sharp increase in the equivalent pressure.

### Empirical correlation for literature data

**Figure 10:**
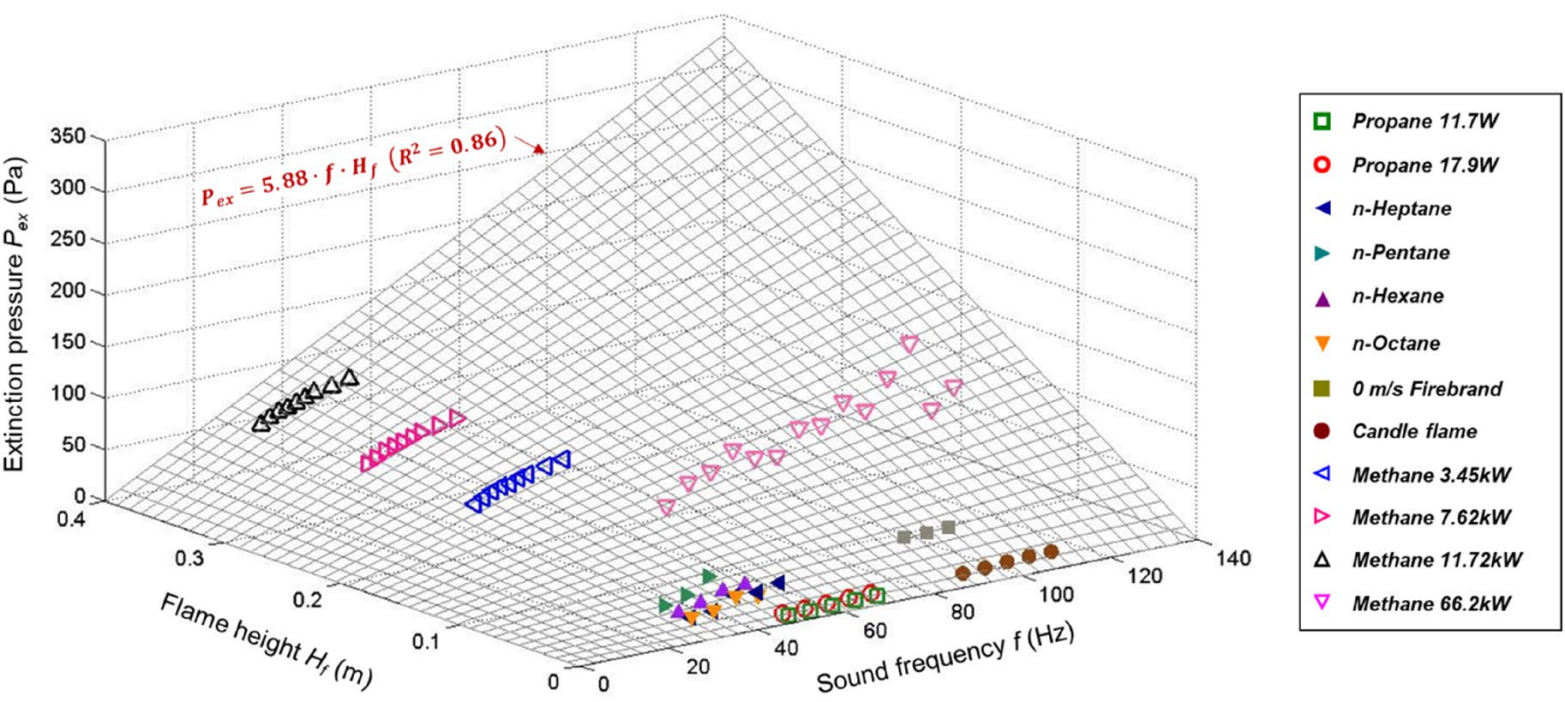
A correlation between local critical sound pressure ${P}_{ex}$, sound frequency $f$, and flame height ${H}_{f}$.

Table 2 summarized the measured the critical local sound pressure (${P}_{ex}$ in Pa) for flame extinction as a function of sound frequency from the literature^6–9,14,15^. The flame-related parameters like the flame type, height, heat release rate (HRR), and Froude number were also listed. Based on Eqs. (3) and (6), an empirical correlation can be proposed for the extinction limits,

11 $${{P}_{ex}} = 5.88 \times f \times {{H}_{f}}$$

which connects the local sound pressure (${P}_{ex}$ in Pa) and frequency ($f$ in Hz) with flame height (${H}_{f}$ in m). The flame height is used here because it is proportional to the critical flame pulsating displacement (see Fig. 4b and Eq. (3)). Note that this empirical correlation is valid for buoyancy-driven flames ($Fr<$ 1) with a frequency ranging from 30–140 Hz. The validity of Eq. (11) is shown in Fig. 10, where the goodness of the fitting is *R*^2^ = 0.86.

**Table 2:** Summary of acoustic extinction limit and the flame parameter, where the unit of ${P}_{ex}$ is Pa.

Fuel	Height [m]	HRR [W]	$Fr$	*f* [Hz]	${P}_{ex}$ [Pa]	Fitting correlation	Source
3 m/s firebrand	0.028	10.0	32.14	95–105	1.5–2.3	${P}_{ex}=0.09f-6.74$	^15^
Propane gas	0.010	11.7	0.003	50–70	1.0–3.1	${P}_{ex}=0.11f-4.5$ 2	This work
3 m/s dripping	0.010	16.5	90	90–110	6.3–8.6	${P}_{ex}=0.13f-3.89$	^14^
Propane gas	0.015	17.9	0.005	50–70	1.2–3.3	${P}_{ex}=0.11f-4.5$ 1	This work
Candle	0.012	20	0.83	90–110	6.4–9.8	${P}_{ex}=0.18f-9.81$	^14^
Methane	0.150	66.2	0.46	50–125	42.7–101.6	${P}_{ex}=1.01f-4.22$	^6^
n-Pentane	0.040	126	0.79	30–50	16.7–36.0	${P}_{ex}=1.93f-42.52$	^7^
n-Octane	0.017	126	0.34	30–50	14.9–22.4	${P}_{ex}=0.62f-3.41$	
n-Hexane	0.028	126	0.54	30–50	14.5–28.6	${P}_{ex}=0.99f-14.68$	
n-Heptane	0.018	126	0.35	30–50	13.6–29.9	${P}_{ex}=0.84f-11.41$	
0 m/s firebrand	0.08	250	0.96	95–105	9.5–10.1	${P}_{ex}=0.07f+3.07$	^15^
Methanol spray	0.40	1,137	24	70–140	13.0–24.1	${P}_{ex}=0.19f-0.76$	^9^
Methane	0.20	3,450	0.08	30–50	50.9–77.1	${P}_{ex}=1.26f+15.88$	^8^
Methane	0.29	7,640	0.28	30–50	53.9–80.6	${P}_{ex}=1.31f+17.07$	
Methane	0.38	11,720	0.52	30–50	56.1–82.9	${P}_{ex}=1.31f+19.66$

In past studies, regardless of the flame type and whether cylindrical tubes were used, the membrane-based speaker was always used as the external sound source. As analyzed above, the acoustic-induced air-molecule displacement is two orders of magnitude smaller than the flame displacement. Therefore, the so-called ‘acoustic-driven flame extinction’ should actually be the ‘membrane-induced flame extinction’. Based on Eqs. (10) and (11), a correlation may be proposed to obtain the equivalent sound pressure (${P}_{eq}$) for a pure acoustic-driven flame extinction as

12 $${{P}_{eq}} \approx 100{{P}_{ex}} = 588 \times f \times {{H}_{f}}$$

Note that depending on the distance between membrane and flame, the ratio of ${\delta }_{m}/\bar{\sigma }$ may change. Thus, this empirical correlation is only a rough estimation for the acoustic-driven extinction limit. Its applicability still needs further verification by experiments and numerical simulations.

## Conclusions

This work experimentally explored the impacts of the sound source on the extinction of laminar diffusion flames. Results showed that once the flame pulsating displacement (${\delta }_{f}$) reaches a critical value (${\delta }_{ex}$), extinction (or blow off) must occur, irrespective of the sound environment used. A critical flame strain rate, as a function of ${\delta }_{ex}$, can characterize the blow-off limit like the classical counterflow flames. Results also confirmed that the sound source with a lower frequency could produce a larger displacement of both membrane and flame and thus show a better flame-extinction performance.

Acoustic analysis of the experimental data confirmed that the flame pulsating displacement is dominated by the airflow caused by the motion of the speaker membrane (or diaphragm), which is around 100 times larger than the displacement of air molecule caused by a pure acoustic wave (longitudinal pressure oscillation). In other words, the so-called "acoustic extinction" is not caused by the sound wave, but by the pulsating wind induced by the speaker (sound source). Thus, an equivalent sound pressure for a pure acoustic-driven extinction seems expected to be two orders of magnitude larger than the literature values. Moreover, an empirical correlation was proposed to estimate the pure acoustic-driven extinction limits for different flame types and sizes. This work improves the understanding of flame dynamics under an external sound field and source; thus, helping establish a scientific framework for acoustic-based fire suppression technologies.

## Supplementary Information

Supplementary Video 1: Fluctuation of a 15-mm flame.

Supplementary Video 2: Extinction of a 15-mm flame.

Supplementary Video 3: Fluctuation of speaker membrane.

## Symbols

*C*: Sonic speed (m/s)
*d**(y,t)*: Local flame deflection (mm)
*Da*: Damköhler number
$\bar{D}$: Flame average displacement (mm)
*f*: Sound frequency (Hz)
*Fr*: Froude number
*g*: Gravitational constant (m/s^2^)
*Gr*: Grashof number
*H*: Height (mm)
$\overrightarrow{k}$: Angular wavevector (rad/m)
*M*: Frame number of videos
*N*: Pixel number on flame image
*P*: Sound pressure (Pa)
$\dot{Q}$: Flow rate (mL/s)
$$\overrightarrow{r}$$: Position vector
*Re*: Reynolds number
*t*: Time (s)
*t*_*0*_: Duration of video (s)
*V*: Velocity (mm/s)
$$\overrightarrow{x}$$: Horizontal direction
$$\overrightarrow{y}$$: Vertical direction
*Z*: Acoustic impedance of air (Pa s/m)

## Greek

$$\alpha$$: Strain rate (s^-1^)
$\delta$: Flame pulsating displacement (mm)
$\rho$: Gas density (kg/m^3^)
$\sigma$: Gas-particle displacement (mm)
$\varphi$: Phase of sound (rad)
$\omega$: Angular frequency (rad/s)

## Subscripts

*0*: Initial
*a*: Air
*ex*: Extinction
*eq*: Equivalence
*f*: Flame
*F*: Fuel
*m*: Speaker membrane
*ref*: Reference value
*s*: Sound
